# Solubility and Thermodynamic Properties of Febuxostat in Various (PEG 400 + Water) Mixtures

**DOI:** 10.3390/ma15207318

**Published:** 2022-10-19

**Authors:** Adel F. Alghaith, Wael A. Mahdi, Nazrul Haq, Sultan Alshehri, Faiyaz Shakeel

**Affiliations:** Department of Pharmaceutics, College of Pharmacy, King Saud University, Riyadh 11451, Saudi Arabia

**Keywords:** dissolution thermodynamics, febuxostat, molecular interactions, {PEG 400 (1) + water (2)} mixtures, solubility

## Abstract

The solubility of the poorly soluble medicine febuxostat (FXT) (3) in various {polyethylene glycol 400 (PEG 400) (1) + water (H_2_O) (2)} mixtures has been examined at 298.2–318.2 K and 101.1 kPa. FXT solubility was measured using an isothermal method and correlated with “van’t Hoff, Apelblat, Buchowski–Ksiazczak *λh*, Yalkowsky–Roseman, Jouyban–Acree, and Jouyban–Acree-van’t Hoff models”. FXT mole fraction solubility was enhanced via an increase in temperature and PEG 400 mass fraction in {(PEG 400 (1) + H_2_O (2)} mixtures. Neat PEG 400 showed the highest mole fraction solubility of FXT (3.11 × 10^–2^ at 318.2 K), while neat H_2_O had the lowest (1.91 × 10^–7^ at 298.2 K). The overall error value was less than 6.0% for each computational model, indicating good correlations. Based on the positive values of apparent standard enthalpies (46.72–70.30 kJ mol^−1^) and apparent standard entropies (106.4–118.5 J mol^−1^ K^−1^), the dissolution of FXT was “endothermic and entropy-driven” in all {PEG 400 (1) + H_2_O (2)} mixtures examined. The main mechanism for FXT solvation in {PEG 400 (1) + H_2_O (2)} mixtures was discovered to be an enthalpy-driven process. In comparison to FXT-H_2_O, FXT-PEG 400 showed the strongest molecular interactions. In conclusion, these results suggested that PEG 400 has considerable potential for solubilizing a poorly soluble FXT in H_2_O.

## 1. Introduction

**Figure 1:**
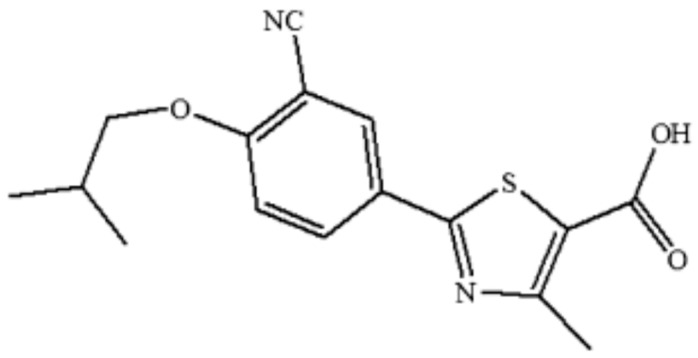
Chemical structure of febuxostat (FXT).

Febuxostat (FXT) (Figure 1) is a selective nonpurine inhibitor of xanthine oxidoreductase [1,2,3]. It is advised for the management of hyperuricemia in gouty individuals [3,4]. One of FXT’s properties is polymorphism [5]. The three polymorphs (forms A, B, and C) and two solvates (BH and D) make up the five distinct forms of FXT [6,7,8]. The preferred form of FXT is Form A, and managing its crystallization process is difficult [7]. Form H, a unique crystalline form of FXT that has been shown to be stable under a number of conditions, is the best form to use for designing dosage forms [1]. FXT is a biopharmaceutical classification system (BCS) class II medicine, which means it shows high permeability and poor aqueous solubility [2]. BCS class II drugs, such as FXT are difficult to formulate due to their extremely poor water (H_2_O) solubility [9]. Different formulation strategies, including the use of microsponges [10], nanosponges [11], polymeric nanoparticles [12], ternary solid dispersion [13], nanoemulsion [14], self-nanoemulsifying formulations [15,16], self-microemulsifying formulation [17], ethosomes [18], and nanostructure lipid carriers [19] have been used to enhance the fundamental and physicochemical characteristics of FXT.

The pharmaceutical industry has acknowledged the importance of solubility expertise over many years [20,21]. The solubility profile of pharmaceuticals, in particular in the field of drug research and development, offers valuable information for enhancing the quality of drug candidates and raises the success rate in a clinic by assisting pharmacists in making knowledgeable judgments [22]. Furthermore, solubility data is useful for estimating in vivo pharmacokinetics, which improves dose prediction [23]. A cosolvency approach has been employed extensively in pharmaceutical science and practice [24]; it is one of many approaches that have been studied over time to increase the solubility of pharmaceuticals [25,26,27,28]. As a consequence, the cosolvency approach using polyethylene glycol 400 (PEG 400) as a cosolvent was used to enhance the solubility of FXT in this study. The solubility data of pharmaceutical compounds are a significant physicochemical feature for a variety of industrial activities, such as purification, manufacturing, formulation development, and other applications [29,30,31]. There is little information on FXT solubilization in mixtures of H_2_O and a cosolvent. FXT solubility in neat H_2_O at 310.2 K has been documented [9]. The literature contains a good report on FXT solubility in four different neat solvents, including methanol, ethanol, acetone, and ethyl acetate, at several temperatures under atmospheric pressure [1]. The solubility of FXT in eleven different pure solvents, including H_2_O, methanol, ethanol, isopropanol, 1-butanol, 2-butanol, ethylene glycol, propylene glycol, PEG 400, Carbitol, and dimethyl sulfoxide, was recently reported by us [32]. FXT solubility in supercritical carbon dioxide at 308–338 K and 100–270 bar has also been documented recently [33].

PEG 400 has been examined as a potential solubilizer/cosolvent for the solubilization of several weakly soluble pharmacological molecules, such as lornoxicam, tenoxicam, dihydropyrimidine derivative, ferulic acid, pterostilbene, amlodipine besylate, and mesalazine [34,35,36,37,38,39,40]. The solubility data and thermodynamic parameters of FXT (3) in binary {PEG 400 (1) + H_2_O (2)} mixtures at various temperatures (298.2–318.2 K) and fixed atmospheric pressure (101.1 kPa) are not reported in the literature. Therefore, in order to ascertain the solubility data and thermodynamic parameters of FXT (3) in various {PEG 400 (1) + H_2_O (2)} combinations, including neat PEG 400 and H_2_O, at 298.2–318.2 K and 101.1 kPa, this investigation was carried out. The development of the study drug’s dosage forms, pre-formulation testing, and purification could all benefit from the data collected during the data gathering phase of this study.

## 2. Materials and Methods

### 2.1. Materials

FXT (form H) was provided by “E-Merck (Darmstadt, Germany)”. PEG 400 was procured from “Sigma Aldrich (St. Louis, MO, USA)”. The Milli-Q unit was used to obtain purified H_2_O. Table 1 provides a summary of each material’s specifics.

### 2.2. Determination of FXT (3) Solubility in {PEG 400 (1) + H_2_O (2)} Mixtures

Utilizing a “Digital Analytical Balance (Mettler Toledo, Greifensee, Switzerland)” with a sensitivity of 0.10 mg, mass measurements of all {PEG 400 (1) + H_2_O (2)} solutions were taken. The mass percentage of PEG 400 varied by 0.10 in a number of {PEG 400 (1) + H_2_O (2)} mixes, ranging from 0.10–0.90. Each {PEG 400 (1) + H_2_O (2)} mixture was taken in triplicate [31]. Utilizing an isothermal method [41], the mole fraction solubility of FXT against mass fraction of PEG 400 (*m* = 0.0 − 1.0; *m* is the mass fraction of PEG 400 in {PEG 400 (1) + H_2_O (2)} mixtures) in binary {PEG 400 (1) + H_2_O (2)} mixtures) and neat PEG 400 and H_2_O was determined at 298.2-318.2 K and 101.1 kPa. In essence, the known amounts of each {PEG 400 (1) + H_2_O (2)} mixture and neat PEG 400 and H_2_O were combined with extra FXT crystals. Three replicates of experiments were run. The resulting samples were saturated in the “WiseBath^®^ WSB Shaking Water Bath (Model WSB-18/30/-45, Daihan Scientific Co. Ltd., Seoul, Korea)” for 72 h in order to reach equilibrium [32]. After reaching equilibrium, the saturated samples were removed from the shaker and centrifuged at 5000rpm for 30 min. A previously described HPLC method at 354 nm was used to measure the FXT concentration after the supernatants were extracted and diluted (as necessary) [32]. FXT mole fraction solubilities (*x*_e_) were computed using Equations (1) and (2):

(1) $$x_{e}=\frac{m_{1}/M_{1}}{m_{1}/M_{1}+m_{2}/M_{2}}$$

(2) $$x_{e}=\frac{m_{1}/M_{1}}{m_{1}/M_{1}+m_{2}/M_{2}+m_{3}/M_{3}}$$

where, *m*_1_ = FXT mass, *m*_2_ = PEG 400 mass, *m*_3_ = H_2_O mass, *M*_1_ = FXT molar mass, *M*_2_ = PEG 400 molar mass, and *M*_3_ = H_2_O molar mass. Equation (1) was used for the computation of FXT *x*_e_ values in neat PEG 400 and H_2_O and Equation (2) was used for the computation of FXT *x*_e_ values in {PEG 400 (1) + H_2_O (2)} mixtures.

### 2.3. Hansen Solubility Parameters (HSPs) of FXT and Various {PEG 400 (1) + H_2_O (2)} Mixtures

A solute’s solubility in a plain solvent or a cosolvent-H_2_O mixture directly relates to its HSP. According to reports, a solute will be most soluble in a certain solvent when its HSP is closed with that of the solvent [42]. The HSPs for the tested drug, FXT; neat PEG 400; and neat H_2_O were consequently computed. For FXT, Equation (3) was used to determine total HSP (*δ*_t_) value [43,44,45,46]:

(3) $$\delta_{t}^{2}=\delta_{d}^{2}+\delta_{p}^{2}+\delta_{h}^{2}$$

where “*δ*_d_ = dispersion HSP; *δ*_p_ = polar HSP, and *δ*_h_ = hydrogen-bonded HSP”. These values for TXT were calculated using “HSPiP software (version 4.1.07, Louisville, KY, USA)” [44]. The *δ*_t_ value for neat H_2_O and neat PEG 400, on the other hand, was derived from the literature [38].

With the help of Equation (4), the HSP for different {PEG 400 (1) + H_2_O (2)} mixtures free of FXT (*δ*_mix_) was computed [46]:

(4) $$\delta_{\mathrm{mix}}=\propto \delta_{1}+\left(1-\propto \right)\delta_{2}$$

where *α* = PEG 400 volume fraction in {PEG 400 (1) + H_2_O (2)} mixture; *δ*_1_ = neat PEG 400 HSP, and *δ*_2_ = neat H_2_O HSP.

### 2.4. FXT Ideal Solubility and Solute–Solvent Interactions

With the help of Equation (5), an “ideal solubility (*x*^idl^)” of FXT at 298.2–318.2 K was calculated [47]:

(5) $$\ln\;x^{\mathrm{idl}}=\frac{-\Delta H_{\mathrm{fus}}\left(T_{\mathrm{fus}}-T\right)}{RT_{\mathrm{fus}}T}+\left(\frac{\Delta C_{p}}{R}\right)[\frac{T_{\mathrm{fus}}-T}{T}+\ln\left(\frac{T}{T_{\mathrm{fus}}}\right)]\;$$

where *T* = absolute temperature; *T*_fus_ = FXT fusion temperature; *R* = universal gas constant; ∆*H*_fus_ = FXT fusion enthalpy, and ∆*C*_p_ = difference in the molar heat capacity of FXT solid state with its liquid state [48]. Equation (6) was used to compute the ∆*C*_p_ for FXT [47,48]:

(6) $$\Delta C_{p}=\frac{\Delta H_{fus}}{T_{fus}}$$

According to Reference [2], the *T*_fus_ and ∆*H*_fus_ values for FXT were 486.53 K and 27.58 kJ mol^−^^1^, respectively. The ∆*C*_p_ for FXT was determined to be 56.68 J mol^−1^ K^−1^ using Equation (6). Finally, the *x*^idl^ values for FXT were calculated using Equation (5). Equation (7) was used to determine the activity coefficient (γ_i_) values for FXT in binary {PEG 400 (1) + H_2_O (2)} mixtures including neat PEG 400 and H_2_O [47,49]:

(7) $$\gamma_{i}=\frac{x^{\mathrm{idl}}}{x_{e}}$$

The chemical basis of solute–solvent interactions was explained using FXT γ_i_ data.

### 2.5. FXT Solubility Correlation Using Computational Models

For useful predictions and validations, computational validation of experimental solubility data of solutes is essential [50,51]. In order to correlate the experimental solubility data of FXT, “van’t Hoff, Apelblat, Buchowski–Ksiazczak *λh*, Yalkowsky-Roseman, Jouyban–Acree, and Jouyban–Acree–van’t Hoff models” were used [38,50,51,52,53,54,55,56,57,58]. MS Excel 2013 was used for all modeling tasks. Using Equation (8), “van’t Hoff model solubility (*x*^van’t^)” of FXT (3) in binary {PEG 400 (1) + H_2_O (2)} mixtures was computed [38]:

(8) $$\ln\;x^{\mathrm{van}\text{’}t}=a+\frac{b}{T}$$

where *a* and *b* are Equation (8) coefficients obtained from the least squares approach [56]. The FXT’s *x*_e_ and *x*^van’t^ values were correlated via “root mean square deviation (*RMSD*)”. The *RMSD* was computed using a formula from the literature [36].

Using Equation (9), the “Apelblat model solubility (*x*^Apl^)” of FXT (3) in binary {PEG 400 (1) + H_2_O (2)} mixtures was computed [52,53]:

(9) $$\ln\;x^{\mathrm{Apl}}=A+\frac{B}{T}+C\ln\left(T\right)$$

where *A*, *B*, and *C* are Equation (9) coefficients determined from the measured FXT solubility data documented in Table 2 using “nonlinear multivariate regression analysis” [36]. The data from FXT’s *x*_e_ and *x*^Apl^ were also correlated by *RMSD*. Using Equation (10), the “Buchowski–Ksiazczak *λh* solubility (*x*^*λh*^)” of FXT (3) in binary {PEG 400 (1) + H_2_O (2)} mixtures was computed [54,55]:

(10) $$\ln\;[1+\frac{\lambda \left(1-x^{\lambda h}\right)}{x^{\lambda h}}]=\lambda h\;[\frac{1}{T}-\frac{1}{T_{\mathrm{fus}}}]\;$$

where *λ* and *h* are Equation (10) adjustable parameters.

Since Equations (8)–(10) describe solubility values at different temperatures in a specific solvent composition, they cannot be utilized to determine the solubility values of a binary solvent combination at different solvent compositions. As a consequence, cosolvency-based models, such as Yalkowsky–Roseman, Jouyban–Acree, and Jouyban–Acree–van’t Hoff models are required for this purpose. Using Equation (11), “logarithmic solubility of the Yalkowsky–Roseman model (log *x*^Yal^)” for FXT (3) in binary {PEG 400 (1) + H_2_O (2)} mixtures was computed [57]:

(11) $$\log x^{\mathrm{Yal}}=w_{1}\log x_{1}+w_{2}\log x_{2}$$

where *x*_1_ = FXT solubility (3) in PEG 400 (1); *x*_2_ = FXT solubility in H_2_O (2); *w*_1_ = PEG 400 mass fraction, and *w*_2_ = H_2_O mass fraction. Equation (11) links the solubility data of solutes in various solvent compositions at a particular temperature.

Equation (12) is used to calculate the solubility of solutes in cosolvent compositions and temperature ($x_{m,T}$) using the “Jouyban–Acree model” [58]:

(12) $$\ln x_{m,T}=w_{1}\ln x_{1,T}+w_{2}\ln x_{2,T}+(\frac{w_{1}.w_{2}}{T})\sum_{i=0}^{2}J_{i}{\left(w_{1}-w_{2}\right)}^{i}$$

where $x_{1,T}$ and $x_{2,T}$ are the solubility of FXT in PEG 400 (1) and H_2_O (2), respectively, at temperature *T*, and *J* terms are Equation (12) parameters. The solubility of FXT in neat PEG 400 and H_2_O are needed as input values to calculate the solubility of FXT in cosolvent compositions at the temperature of interest. To get around this restriction, Equations (8) and (12) can be coupled to form the “Jouyban–Acree–van’t Hoff model” [58].

### 2.6. Thermodynamic Parameters

At the mean harmonic temperature (*T*_hm_), all apparent thermodynamic parameters for FXT were measured [47]. Using the formula from the literature [47,58], the *T*_hm_ was determined. *T*_hm_ for FXT was determined to be 308 K. An apparent thermodynamic analysis was used to calculate a number of apparent thermodynamic parameters. The “van’t Hoff and Gibbs equations” were applied for this purpose. Equation (13), applied with *T*_hm_ = 308 K, was used to calculate the apparent standard enthalpy (Δ_sol_*H*^0^) data for FXT (3) in various {PEG 400 (1) + H_2_O (2)} mixtures [47,59]:

(13) ∂ln xe∂1T−1ThmP=−ΔsolH0R

**Figure 2:**
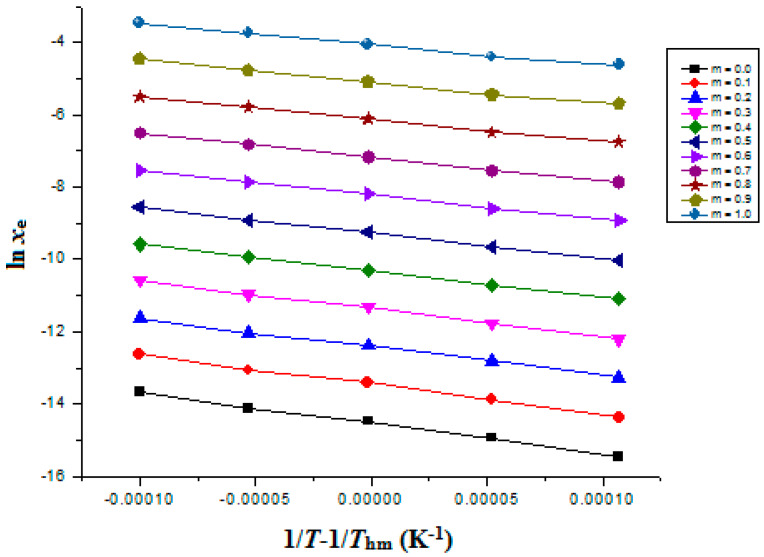
van’t Hoff curves for FXT graphed between ln *x*_e_ and 1/*T*-1/*T*_hm_ for FXT in various {PEG 400 (1) + H_2_O (2)} mixtures.

The graphed “van’t Hoff” curves between the ln *x*_e_ values of FXT and 1T−1Thm were used to calculate the “Δ_sol_*H*^0^” for FXT. Figure 2 includes the van’t Hoff curves for FXT (3) in binary {PEG 400 (1) + H_2_O (2)} mixtures.

Additionally, using the Krug et al. approach and Equation (14) [59], the apparent standard Gibbs energy (Δ_sol_*G*^0^) for FXT (3) in binary {PEG 400 (1) + H_2_O (2)} mixtures was calculated at *T*_hm_ = 308 K:

(14) $$\Delta_{\mathrm{sol}}G^{0}=-RT_{\mathrm{hm}}\times \mathrm{intercept}\;$$

where the intercept values for FXT (3) in binary {PEG 400 (1) + H_2_O (2)} mixtures were derived from “van’t Hoff plots” presented in Figure 2.

Using Equation (15), the apparent standard entropy (Δ_sol_*S*^0^) for FXT (3) in binary {PEG 400 (1) + H_2_O (2)} mixtures was computed [47,59,60]:

(15) $$\;\Delta_{\mathrm{sol}}S^{0}=\frac{\Delta_{\mathrm{sol}}H^{0}-\Delta_{\mathrm{sol}}G^{0}}{T_{\mathrm{hm}}}\;$$

### 2.7. Enthalpy–Entropy Compensation Analysis

An enthalpy-entropy compensation analysis is utilized, as previously reported [38], to examine the solvation behavior of FXT (3) in binary mixtures of {PEG 400 (1) + H_2_O (2)}. The weighted graphs of Δ_sol_*H*° vs. Δ_sol_*G*° were created for this analysis at *T*_hm_ = 308 K [39,40].

## 3. Results and Discussion

### 3.1. Measured Solubility Data of FXT

**Figure 3:**
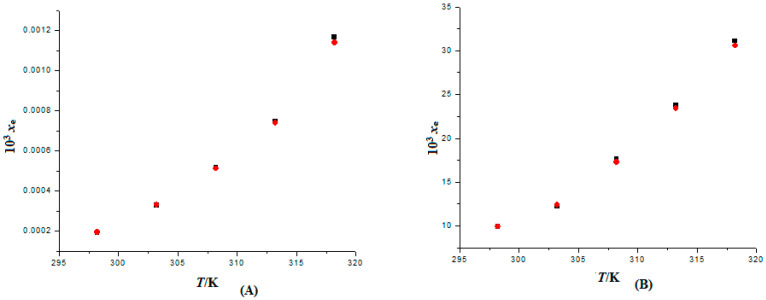
Graphical comparison of FXT mole fraction solubility data in (**A**) neat H_2_O and (**B**) neat PEG 400 with those found in the literature at 298.2–318.2 K. The symbol 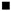 denotes the measured mole fraction solubilities of FXT in (**A**) neat H_2_O and (**B**) neat PEG 400, and the symbol 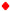 denotes the reported solubilities of FXT in (**A**) neat H_2_O and (**B**) neat PEG 400 taken from reference [32].

To look into the potential conversion of FXT into polymorphs or solvates/hydrates, the solid phase characterization of FXT before solubility measurement (pure FXT) and after solubility measurement (equilibrated FXT recovered from H_2_O) was conducted. Our most recent work [32] reports the results of this characterization on pure and equilibrated FXT utilizing Fourier transforms infrared spectroscopy (FTIR) and X-ray diffraction (XRD) analyses. In our most recent publication [32], it was discovered that the FTIR and XRD spectra of pure and equilibrated FXT were identical and shared similar peak characteristics. Additionally, no other FTIR or XRD peaks were seen in the equilibrated FXT sample. These findings showed that FXT did not change into polymorphs or solvates/hydrates. The measured solubility data of FXT (3) in binary {PEG 400 (1) + H_2_O (2)} mixtures at five different temperatures and fixed pressure are documented in Table 2. FXT solubility (3) in binary {PEG 400 (1) + H_2_O (2)} mixtures has not been reported. However, the solubility of FXT in neat PEG 400 and H_2_O has been documented. At 310.2 K, the equilibrium solubility of FXT in H_2_O has been reported to be 10.8 mg L^−1^ (equivalent to 6.15 × 10^−7^ in mole fraction) [9,32]. In this work, the FXT mole fraction solubility was not directly calculated at 310.2 K. However, the interpolation of the curve drawn between ln *x*_e_ and 1/*T* revealed that the FXT mole fraction solubility at 310.2 K was 6.19 × 10^−7^. The recorded value was fairly close to the FXT in H_2_O stated value [9]. FXT mole fraction solubility values in neat PEG 400 and H_2_O at 298.2–318.2 K have also been documented [32]. The measured and reported solubility values of FXT in neat H_2_O and PEG 400 at 298.2–318.2 K are graphically compared in Figure 3. A good correlation was found between the observed solubility values of FXT in neat H_2_O and PEG 400 and those described in the literature, as seen in the results summarized in Figure 3 [32]. These results showed that the measured solubility statistics for FXT were in good agreement with the literature that has been published [9,32]. FXT solubility has also been improved using different techniques, such as solid dispersion, nanomatrix, and nanoemulsion approaches [61,62,63]. The comparative solubility data of FXT in PEG 400 with reported solubility approaches are summarized in Table 3. The equilibrium solubility of FXT using nanomatrix and solid dispersion approaches has been reported as 92.91 and 632.0 µg mL^−1^, respectively [61,62]. The equilibrium solubility of FXT in PEG 400 was 7053 µg mL^−1^ in this work, which was approximately 76- and 11-fold higher than its reported solubility in nanomatrix and solid dispersion, respectively. Reddy and Sundari reported the equilibrium solubility of FXT using solid dispersion and nanoemulsion approaches as 1146 and 655.0 µg mL^−1^, respectively [63]. The equilibrium solubility of FXT in PEG 400 was approximately 6 and 11-folds higher than its reported solubility in solid dispersion and nanoemulsion, respectively. These outcomes suggested the potential of cosolvency approach in solubilization of FXT over other reported approached of FXT solubilization.

**Figure 4:**
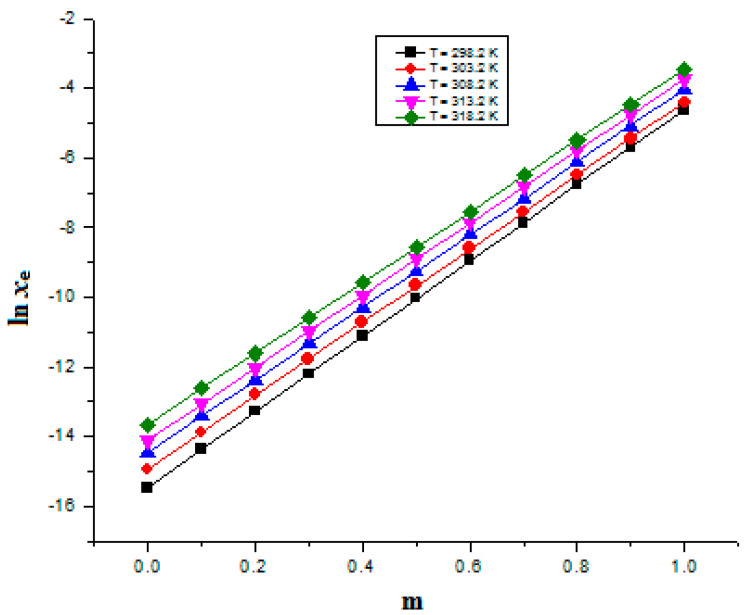
Influence of PEG 400 mass fraction (*m*) on FXT solubility values at 298.2–318.2 K.

In general, it was found that the mole fraction solubility of FXT was lowest in neat H_2_O and highest in neat PEG 400. The low polarity of PEG 400 in contrast to the high polarity of H_2_O may help to explain the maximal solubility of FXT in neat PEG 400 [36,37,38]. When the temperature and PEG 400 mass fraction were increased, FXT (3) mole fraction solubility in various {PEG 400 (1) + H_2_O (2)} mixtures rose. At 298.2–318.2 K, the effect of PEG 400 mass fraction on FXT logarithmic mole fraction solubility was also examined. The results are shown in Figure 4. FXT solubility increased linearly with an increase in PEG 400 mass fraction in mixes of {PEG 400 (1) + H_2_O (2)} at each temperature under investigation. FXT mole fraction solubility rose significantly from neat H_2_O to neat PEG 400. PEG 400 might therefore be used as a solubilizer in the solubilization of FXT in an aqueous medium.

### 3.2. Determination of HSPs

Using “HSPiP software”, the *δ*_t_ for FXT was calculated to be 21.70 MPa^1/2^, confirming that FXT had low polarity. According to the published research, the HSP values for neat PEG 400 (*δ*_1_) and neat H_2_O (*δ*_2_) are 18.90 and 47.80 MPa^1/2^, respectively [38]. According to the calculations, the *δ*_mix_ values for various {PEG 400 (1) + H_2_O (2)} mixtures free of FXT ranged from 21.79 to 44.91 MPa^1/2^. In mixtures of {PEG 400 (1) + H_2_O (2)}, it was discovered that the *δ*_mix_ values decreased as the PEG 400 mass fraction increased. The highest and lowest *δ*_mix_ values were consequently measured at *m* = 0.1 and *m* = 0.9, respectively. However, it was discovered that a decrease in *δ*_mix_ values improved the solubility values of FXT. Generally speaking, the HSP for neat PEG 400 (*δ*_1_ = 18.90 MPa^1/2^) and FXT (*δ*_t_ = 21.70 MPa^1/2^) were comparable. FXT solubility in neat PEG 400 was likewise discovered to be the highest during the investigations. As a result, the FXT solubility data obtained from experiments using mixes of {PEG 400 (1) + H_2_O (2)} closely matched these results.

### 3.3. Determination of Ideal Solubility and Solute–Solvent Interactions

The values of FXT’s *x*^idl^ are documented in Table 2. FXT’s *x*^idl^ values were determined to be 3.55 × 10^−2^ to 5.52 × 10^−2^ between 298.2 and 318.2 K. In neat H_2_O, FXT had much higher *x*^idl^ values than *x*_e_ values. FXT’s *x*^idl^ values, however, were nearly identical to its *x*_e_ values in neat PEG 400 at all study temperatures. It can be used as the optimum cosolvent for FXT solubilization because neat PEG 400 has the highest solubility of FXT.

The *γ*_i_ values for FXT in various {PEG 400 (1) + H_2_O (2)} mixtures at five different temperatures are documented in Table 4. The *γ*_i_ value of FXT was greatest in neat H_2_O at all studied temperatures. However, in neat PEG 400, the *γ*_i_ of FXT was lowest at each temperature examined. The *γ*_i_ values for FXT were noticeably lower for neat PEG 400 than for neat H_2_O. The largest *γ*_i_ for FXT in neat H_2_O may be explained by the least solubility of FXT in that solution. These results suggest that when compared to FXT-H_2_O combination, FXT-PEG 400 combination has the greatest number of solute–solvent interactions at the molecular level.

### 3.4. FXT Solubility Correlation Using Computational Models

Six different mathematical models, including “van’t Hoff, Apelblat, Buchowski–Ksiazczak *λh*, Yalkowsky-Roseman, Jouyban–Acree, and Jouyban–Acree–van’t Hoff models” were used to correlate FXT’s solubility data [38,50,51,52,53,54,55,56,57,58]. The results for the correlation using the “van’t Hoff model” are documented in Table 5. This model’s overall *RMSD* was computed to be 2.30%. The determination coefficient (*R*^2^) for FXT was computed to be 0.9941–0.9998 in all cosolvent mixtures and in neat PEG 400 and H_2_O. The “van’t Hoff model” findings and FXT (3) measured solubility data in {PEG 400 (1) + H_2_O (2)} mixtures demonstrated a good correlation.

**Figure 5:**
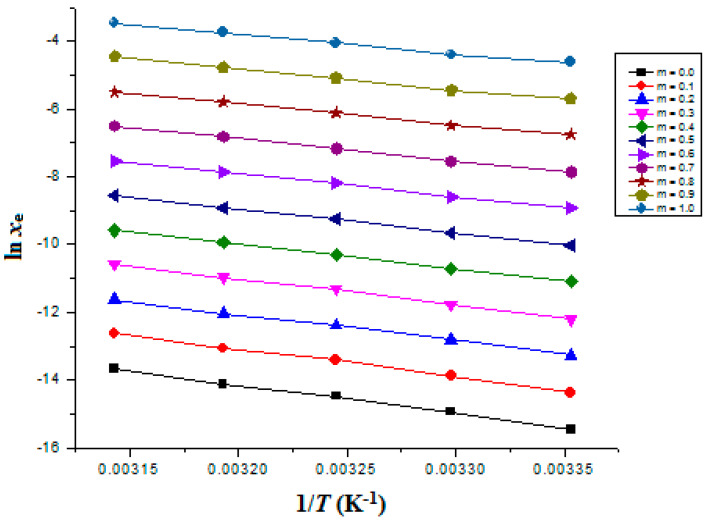
Correlation of experimental FXT (3) solubilities with the “Apelblat model” in various {PEG 400 (1) + H_2_O (2)} mixtures as a function of 1/*T*; symbols denote the experimental FXT solubility data, whereas solid lines denote the “Apelblat model” FXT solubility data.

Figure 5 provides a graphical correlation of measured and Apelblat solubility data of FXT in binary solvent mixtures and in neat H_2_O and PEG 400. The results summarized in Figure 5 showed a good correlation of measured solubility values of FXT with “Apelblat model”. The model parameters and correlation outcomes of FXT in binary {PEG 400 (1) + H_2_O (2)} mixtures with the “Apelblat model” are documented in Table 6. This model’s overall *RMSD* was computed to be 2.06%. FXT (3) showed a *R*^2^ of 0.9952–0.9997 in all cosolvent mixtures, including neat PEG 400 and H_2_O. The outcomes of the “Apelblat model” and FXT (3) measured solubility data in binary {PEG 400 (1) H_2_O (2)} mixtures also demonstrated a significant correlation.

Table 7 documents the outcomes of “Buchowski–Ksiazaczak *λh*” results for FXT in cosolvent mixtures and neat solvents. This model’s overall *RMSD* was computed to be 4.68%. These outcomes also showed a good correlation of FXT’s measured solubility data with “Buchowski–Ksiazaczak *λh* model”.

Table 8 documents the outcomes of the correlation with the “Yalkowsky-Roseman model”. The overall *RMSD* for this model was calculated to be 5.21%, demonstrating a strong correlation of FXT (3) solubility data in various {PEG 400 (1) + H_2_O (2)} combinations using the “Yalkowsky-Roseman model”.

FXT (3) solubility was also linked to “Jouyban–Acree and Jouyban–Acree–van’t Hoff models” [55] in several {PEG 400 (1) + H_2_O (2)} combinations at several temperatures and solvent compositions. Table 9 documents the outcomes of the correlation with the “Jouyban–Acree and Jouyban–Acree–van’t Hoff models”. The overall *RMSDs* for the “Jouyban–Acree and Jouyban–Acree–van’t Hoff models” were computed to be 0.98% and 1.09%, respectively.

### 3.5. Apparent Thermodynamic Parameters for FXT Dissolution

The Δ_sol_*H°* values for FXT in all cosolvent compositions and neat PEG 400 and H_2_O, were derived using the van’t Hoff methodology. Figure 2 presents the linear van’t Hoff plots of FXT in all cosolvent mixtures and neat PEG 400 and H_2_O where R^2^ > 0.99 was obtained, as mentioned in Table 10. Table 10 also documents the results for all thermodynamic parameters. FXT (3) Δ_sol_*H°* values varied from 46.72 to 70.30 kJ mol^–1^ in several {PEG 400 (1) + H_2_O (2)} mixtures and neat PEG 400 and H_2_O. FXT (3) Δ_sol_*G°* values varied from 10.37 to 37.20 kJ mol^–1^ in several {PEG 400 (1) + H_2_O (2)} mixtures and neat PEG 400 and H_2_O. These outcomes of Δ_sol_*H°* and Δ_sol_*G°* for FXT demonstrated “endothermic dissolution” of FXT (3) in various {PEG 400 (1) + H_2_O (2)} mixtures and neat PEG 400 and H_2_O [37,38]. Various {PEG 400 (1) + H_2_O (2)} combinations and neat PEG 400 and H_2_O were found to have FXT (3) Δ_sol_*S°* values between 106.4 and 118.5 J mol^–1^ K^–1^, suggesting entropy-driven FXT (3) dissolution in these binary mixtures [37]. The dissolution of FXT (3) was discovered to be “endothermic and entropy-driven” in all {PEG 400 (1) + H_2_O (2)} combinations, including neat PEG 400 and H_2_O [37,38].

### 3.6. Enthalpy–Entropy Compensation Analysis

**Figure 6:**
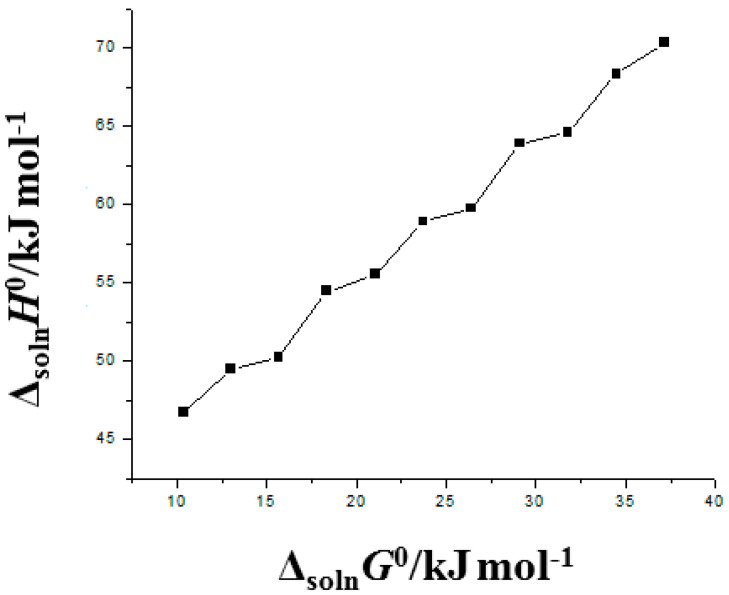
Δ_sol_*H°* vs. Δ_sol_*G°* enthalpy–entropy compensation graph for solubility of FXT in various {PEG 400 (1) + H_2_O (2)} mixtures at *T*_hm_ = 308 K.

The solvation behavior of FXT (3) in several {PEG 400 (1) + H_2_O (2)} combinations and neat PEG 400 and H_2_O was examined using an enthalpy–entropy compensation analysis, and results are documented in Figure 6. Figure 6 shows that in all {PEG 400 (1) + H_2_O (2)} mixtures with neat PEG 400 and H_2_O, FXT (3) offers a nonlinear Δ_sol_*H*° vs. Δ_sol_*G*° trend with a slope greater than 1.0 and an *R*^2^ less than 0.99. Based on these findings, it is believed that the main mechanism for FXT (3) solvation in all {PEG 400 (1) + H_2_O (2)} combinations as well as in neat PEG 400 and H_2_O is enthalpy-driven. This method of FXT solvation should be explained by the fact that FXT solvates better in neat PEG 400 molecules than in neat H_2_O molecules [37,38]. As a result, FXT-PEG 400 molecules interacted with one another more strongly than FXT-H_2_O molecules did. In various {PEG 400 (1) + H_2_O (2)} combinations and in neat PEG 400 and H_2_O, FXT (3) solvated similarly to lornoxicam, tenoxicam, dihydropyrimidine derivative, ferulic acid, and pterostilbene [34,35,36,37,38].

The main drawback of this study is that just one set of {PEG 400 (1) + H_2_O (2)} combinations was used to measure the solubility and physicochemical parameters of FXT. The pharmaceutical and chemical industries, however, may be able to use the research’s published results to aid in the purification, recrystallization, pre-formulation testing, and development of the study drug’s dosage form. In the future, a wide range of H_2_O-cosolvent mixes at different temperatures can be used to conduct FXT solubility experiments.

## 4. Conclusions

The solubility of FXT in several {PEG 400 (1) + H_2_O (2)} combinations is not documented in the literature. In this study, FXT (3) solubility in binary {PEG 400 (1) + H_2_O (2)} combinations and neat PEG 400 and H_2_O was tested at various temperatures and atmospheric pressure. With an increase in temperature and PEG 400 mass fraction in all {PEG 400 (1) + H_2_O (2)} combinations, including neat PEG 400 and H_2_O, FXT (3) mole fraction solubilities rose. The highest and lowest solubilities of FXT were found in neat PEG 400 and neat H_2_O, respectively, at each temperature examined. Experimentally recorded FXT (3) solubility data were strongly correlated using six distinct computational models in all {PEG 400 (1) + H_2_O (2)} combinations including neat PEG 400 and H_2_O. All thermodynamic quantities, including Δ_sol_*H°*^,^ Δ_sol_*G°*, and Δ_sol_*S°* in different {PEG 400 (1) + H_2_O (2)} combinations and neat PEG 400 and H_2_O were found to be positive, demonstrating “endothermic and entropy-driven” FXT dissolution. The main mechanism for FXT solvation was enthalpy-driven in all {PEG 400 (1) + H_2_O (2)} mixtures and in neat PEG 400 and H_2_O. The obtained data of this study could be useful in the development of the studied drug’s dosage forms, pre-formulation testing, and purification.

## Figures and Tables

**Table 1:** Materials properties.

Material	Molecular Formula	Molar Mass (g mol^−1^)	CAS RN	Purification Method	Mass Fraction Purity	Analysis Method	Source
FXT	C_16_H_16_N_2_O_3_S	316.40	144060-53-7	None	˃0.99	HPLC	E-Merck
PEG 400	H(OCH_2_CH_2_)_n_OH	400.00	25322-68-3	None	˃0.99	HPLC	Sigma Aldrich
Water	H_2_O	18.07	7732-18-5	None	-	-	Milli-Q

FXT: febuxostat; PEG 400: polyethylene glycol 400; HPLC: high-performance liquid chromatography.

**Table 2:** Measured mole fraction solubility (*x*_e_) and ideal solubility (*x*^idl^) data of FXT (3) in binary {PEG 400 (1) + H_2_O (2)} mixtures at 298.2–318.2 K and 101.1 kPa ^a^.

*m* ^a^	*x*_e_^b^				
	*T* = 298.2 K	*T* = 303.2 K	*T* = 308.2 K	*T* = 313.2 K	*T* = 318.2 K
0.0	1.91 × 10^–7^	3.27 × 10^–7^	5.18 × 10^–7^	7.46 × 10^–7^	1.17 × 10^–6^
0.1	5.73 × 10^–7^	9.44 × 10^–7^	1.51 × 10^–6^	2.14 × 10^–6^	3.31 × 10^–6^
0.2	1.72 × 10^–6^	2.75 × 10^–6^	4.22 × 10^–6^	5.98 × 10^–6^	9.01 × 10^–6^
0.3	5.03 × 10^–6^	7.74 × 10^–6^	1.24 × 10^–5^	1.71 × 10^–5^	2.55 × 10^–5^
0.4	1.52 × 10^–5^	2.26 × 10^–5^	3.41 × 10^–5^	4.79 × 10^–5^	6.91 × 10^–5^
0.5	4.42 × 10^–5^	6.43 × 10^–5^	9.59 × 10^–5^	1.36 × 10^–4^	1.96 × 10^–4^
0.6	1.32 × 10^–4^	1.87 × 10^–4^	2.80 × 10^–4^	3.84 × 10^–4^	5.33 × 10^–4^
0.7	3.88 × 10^–4^	5.29 × 10^–4^	7.77 × 10^–4^	1.10 × 10^–3^	1.51 × 10^–3^
0.8	1.18 × 10^–3^	1.52 × 10^–3^	2.21 × 10^–3^	3.05 × 10^–3^	4.08 × 10^–3^
0.9	3.40 × 10^–3^	4.32 × 10^–3^	6.24 × 10^–3^	8.52 × 10^–3^	1.16 × 10^–2^
1.0	9.88 × 10^–3^	1.22 × 10^–2^	1.76 × 10^–2^	2.38 × 10^–2^	3.11 × 10^–2^
*x* ^idl^	3.55 × 10^–2^	3.97 × 10^–2^	4.44 × 10^–2^	4.96 × 10^–2^	5.52 × 10^–2^

^a^ The uncertainties *u* are *u*(*T*) = 0.20 K, *u*(*m*) = 0.0007, and *u*(*p*) = 3 kPa. ^b^ The relative uncertainty *u*_r_ in solubility is *u*_r_(*x*_e_) = 0.03.

**Table 3:** Comparison of current solubility data of FTX in PEG 400 with different approaches of FXT solubility enhancement at 298.2 K.

Solubility Approach	Solubility (µg mL^−1^)	Reference
Nanomatrix	92.91	[61]
Solid dispersion	632.0	[62]
Solid dispersion	1146	[63]
Nanoemulsion	655.2	[63]
PEG 400	7053	Present work

**Table 4:** Activity coefficients (*γ_i_*) of FXT in different {PEG 400 (1) + H_2_O (2)} mixtures at 298.2–318.2 K.

*m*	*γ*_i_				
	*T* = 298.2 K	*T* = 303.2 K	*T* = 308.2 K	*T* = 313.2 K	*T* = 318.2 K
0.0	186,185	121,493	85,826.5	665,33.4	47,365.2
0.1	61,995.4	42,125	29,434.9	23,186.9	16,728.8
0.2	20,595.7	14,491.3	10,530.9	8295.58	6137.04
0.3	7064.03	5138.69	3595.31	2906.06	2165.08
0.4	2336.21	1762.23	1304.59	1035.92	800.167
0.5	802.759	618.725	463.792	364.386	281.921
0.6	269.068	213.120	158.860	129.281	103.713
0.7	91.5310	75.2345	57.2032	45.1986	36.6356
0.8	30.0797	26.2123	20.0819	16.2481	13.5365
0.9	10.4457	9.21501	7.12575	5.82733	4.76068
1.0	3.59131	3.25024	2.52160	2.08352	1.77697

**Table 5:** Results for the van’t Hoff model for FXT (3) in several {PEG 400 (1) + H_2_O (2)} combinations.

*m*	*a*	*b*	*R* ^2^	Overall *RMSD* (%)
0.0	12.880	–8445.1	0.9972	
0.1	13.190	–8210.6	0.9977	
0.2	12.766	–7758.0	0.9987	
0.3	13.549	–7674.4	0.9979	
0.4	12.980	–7177.6	0.9995	
0.5	13.700	–7076.7	0.9998	2.30
0.6	13.430	–6669.2	0.9990	
0.7	14.062	–6541.4	0.9987	
0.8	13.467	–6035.9	0.9963	
0.9	14.224	–5948.0	0.9956	
1.0	14.164	–5611.6	0.9941	

**Table 6:** Results for the Apelblat model for FXT (3) in several {PEG 400 (1) + H_2_O (2)} combinations.

*m*	*A*	*B*	*C*	*R^2^*	Overall *RMSD* (%)
0.0	715.04	–40,696	–104.25	0.9980	
0.1	579.83	–34,242	–84.134	0.9981	
0.2	391.07	–25,144	–56.167	0.9989	
0.3	372.45	–24,169	–53.285	0.9980	
0.4	159.24	–13,912	–21.708	0.9995	
0.5	–82.030	–2704.3	14.228	0.9997	2.06
0.6	148.11	–12,870	–19.990	0.9989	
0.7	–232.67	4762.9	36.652	0.9989	
0.8	–424.68	14,055	65.078	0.9970	
0.9	–695.38	26,602	105.39	0.9976	
1.0	–500.92	18,012	76.502	0.9952	

**Table 7:** Buchowski–Ksiazaczak *λh* model results for FXT (3) in different {PEG 400 (1) + H_2_O (2)} mixtures.

*m*	*λ*	*h*	Overall *RMSD* (%)
0.0	3.4715	2432.6	
0.1	2.6856	3057.3	
0.2	2.1792	3560.1	
0.3	1.2242	6269.0	
0.4	0.77310	9283.7	
0.5	0.15470	45745	4.68
0.6	0.72260	9229.4	
0.7	0.38320	17,070	
0.8	0.06130	98,464	
0.9	0.99860	5956.3	
1.0	1.6304	4441.8	

**Table 8:** Results for Yalkowsky–Roseman model for FXT (3) in several {PEG 400 (1) + H_2_O (2)} combinations at 298.2–318.2 K.

*m*	log *x*^Yal^					Overall *RMSD* (%)
	*T* = 298.2 K	*T* = 303.2 K	*T* = 308.2 K	*T* = 313.2 K	*T* = 318.2 K	
0.1	–6.24	–6.02	–5.83	–5.67	–5.48	
0.2	–5.77	–5.57	–5.37	–5.22	–5.04	
0.3	–5.30	–5.11	–4.92	–4.77	–4.60	
0.4	–4.83	–4.65	–4.47	–4.32	–4.16	5.21
0.5	–4.36	–4.19	–4.01	–3.87	–3.71	
0.6	–3.89	–3.74	–3.56	–3.42	–3.27	
0.7	–3.41	–3.28	–3.11	–2.97	–2.83	
0.8	–2.94	–2.82	–2.65	–2.52	–3.39	
0.9	–2.47	–2.36	–2.20	–2.07	–1.94	

**Table 9:** Results for “Jouyban–Acree” and “Jouyban–Acree–van’t Hoff” models for FXT (3) in different {PEG 400 (1) + H_2_O (2)} mixtures.

System	Jouyban–Acree	Jouyban–Acree–van’t Hoff
		*A*_1_ 14.164
{PEG 400 (1) + H_2_O (2)}	*J*_i_ 11595	*B*_1_ –5611.6
		*A*_2_ 12.880
		*B*_2_ –8445.1
		*J*_i_ 10894
*RMSD* (%)	0.98	1.09

**Table 10:** Apparent thermodynamic parameters (Δ_sol_*H*^0^, Δ_sol_*G*^0^, and Δ_sol_*S*^0^) along with *R*^2^ values for FXT (3) in different {PEG 400 (1) + H_2_O (2)} mixtures ^c^.

*m*	Δ_sol_*H*^0^/kJ mol^−1^	Δ_sol_*G*^0^/kJ mol^−1^	Δ_sol_*S*^0^/J mol^−1^ K^−1^	*R* ^2^
0.0	70.30	37.20	107.4	0.9971
0.1	68.35	34.44	109.9	0.9976
0.2	64.58	31.80	106.4	0.9987
0.3	63.89	29.09	112.9	0.9978
0.4	59.75	26.42	108.1	0.9995
0.5	58.91	23.74	114.1	0.9998
0.6	55.52	21.04	111.9	0.9990
0.7	54.46	18.36	117.1	0.9988
0.8	50.25	15.68	112.2	0.9964
0.9	49.52	13.01	118.5	0.9958
1.0	46.72	10.37	117.9	0.9942

^c^ The relative uncertainties are *u*(Δ_sol_*H*^0^) = 0.013, *u*(Δ_sol_*G*^0^) = 0.037 and *u*(Δ_sol_*S*^0^) = 0.003.

## Data Availability

This study did not report any data.
